# miR-1254 inhibits cell proliferation, migration, and invasion by down-regulating Smurf1 in gastric cancer

**DOI:** 10.1038/s41419-018-1262-x

**Published:** 2019-01-10

**Authors:** Mingkun Jiang, Liang Shi, Chao Yang, Yugang Ge, Linling Lin, Hao Fan, Yu He, Diancai Zhang, Yongchang Miao, Li Yang

**Affiliations:** 1 Department of General Surgery, The First Affiliated Hospital of Nanjing Medical University, Nanjing, China; 2 Department of Liver Surgery/Liver Transplantation Center, The First Affiliated Hospital of Nanjing Medical University, Nanjing, China; 3 Key Laboratory on Living Donor Liver Transplantation, National Health and Family Planning Commission of China, Nanjing, China; 4 Department of General Surgery, the second People’s Hospital of Lianyungang, Lianyungang, China

## Abstract

Gastric cancer (GC) is one of the most frequent malignancies, and increasing evidence supports the contribution of microRNA (miRNAs) to cancer progression. miR-1254 has been confirmed to participate in the regulation of various cancers, while the function of miR-1254 in GC remains unknown. In this study, we investigated the role of miR-1254 in GC. The expression of miR-1254 was detected in human GC specimens and cell lines by miRNA RT-PCR. The effects of miR-1254 on GC proliferation were determined by CCK-8 proliferation assays, colony formation assays, 5-ethynyl-2′-deoxyuridine (EdU) incorporation, and cell-cycle assays. The ability of migration and invasion was examined by transwell and wound-healing assay. Dual Luciferase reporter assay was used to validate the interaction of miR-1254 with its target gene. The xenograft mouse models were conducted to investigate the effects of miR-1254 in vivo. The signaling pathways and epithelial–mesenchymal transition (EMT)-related proteins were detected with western blot. The results showed that miR-1254 inhibited the proliferation, migration and invasion in vitro and suppressed tumorigenesis in vivo. Smurf1 was shown to be the direct target of miR-1254. Overexpressing Smurf1 could partially counteract the effects caused by miR-1254. Similarly, the effects of the miR-1254-inhibitor were also rescued by Smurf1-shRNA. Furthermore, we found that miR-1254 inhibited EMT and decreased the PI3K/AKT signaling pathway through downregulating Smurf1. In summary, overexpression of miR-1254 could suppress proliferation, migration, invasion, and EMT via PI3K/AKT signaling pathways by downregulation of Smurf1 in GC, which suggests a potential therapeutic target for GC.

## Introduction

Gastric cancer (GC) is one of the most frequent malignancies, particularly in Eastern Asia, its incidence and mortality rank the fourth and the third, respectively, in the world^1^. In 2015, estimated 679,100 new GC cases and 498,000 deaths occurred in China^2^. Despite clinical outcome of GC has been gradually improved by early diagnosis, surgical techniques and postoperative chemotherapy, the 5-year survival rate of advanced GC patients is low^3^. Therefore, it is essential to elucidate the molecular mechanisms underlying the development and progression of GC.

MicroRNAs (miRNAs) are a class of evolutionary conserved, small noncoding RNAs consisting of 18–25 nucleotides, which downregulate target mRNAs expression by binding to the 3′-untranslated regions (3′-UTR), leading to suppression of translation or mRNAs degradation^4,5^. The first miRNA was discovered as a small RNA transcribed from the *Caenorhabditis elegans* lin-4 locus in 1993^6^, and mammalian miRNA (let-7) was identified for the first time in 2000^7^. So far, miRNAs have been described as playing an important role in the progression of cancer, such as tumor proliferation, invasion, and metastasis^8^. Dysregulation of miRNAs expression promotes the development of cancer due to the activation of oncogenes and silence of tumor-suppressor genes^9,10^. Accumulating evidence has revealed that miR-1254 might strongly correlate to human cancer, such as non-small-cell lung carcinoma, thyroid cancer, and colorectal cancer^11–13^. However, the biological functions and molecular mechanisms of miR-1254 in GC have not been reported. In this study, we found that miR-1254 inhibited the progression of GC both in vitro and in vivo.

Smad ubiquitin regulatory factor 1 (Smurf1), a C2-WW-HECT ubiquitin ligase, is involved in a variety of biological processes, such as bone homeostasis, embryogenesis, and viral autophagy^14–16^. Moreover, an increasing body of evidence has revealed that Smurf1 exerts a promoting effect in carcinogenesis by regulating downstream proteins^17,18^. Previous studies revealed that Smurf1 as a cancer-related gene could promote EMT and positively regulate the PI3K/AKT signaling pathway, which influenced cancer cell proliferation, migration, and invasion^19^. Bioinformatics analysis and relevant functional assay were used to confirm that Smurf1 was a putative direct target of miR-1254 and played a crucial role in human GC.

In this study, we aimed to investigate the role of miR-1254 in GC and the relation to Smurf1. Our results indicated that overexpressed miR-1254 could inhibit the development and progression of GC by targeting Smurf1 through PI3K/AKT signaling pathways in vitro and in vivo. These findings also provided a basis for miR-1254 as a potential therapeutic target for GC.

## Results

### MiR-1254 is down-regulated in human GC tissues and cell lines

**Fig. 1:**
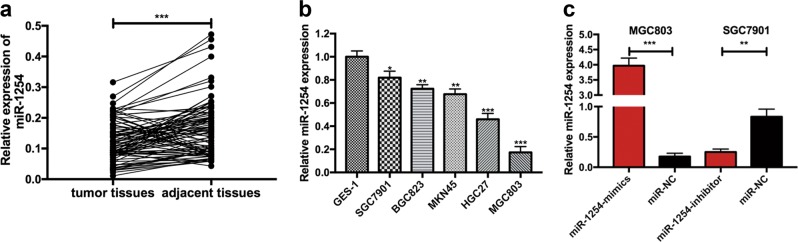
The expression of miR-1254 in GC tissues, GC cells, and transfected cells. **a** The expression levels of miR-1254 in 90 pairs of human GC tissues and adjacent normal tissues were detected using miRNA RT-PCR. **b** The expression levels of miR-1254 in GC cells and GES-1 were examined by miRNA RT-PCR. **c** The efficacy of transfection was verified in cells transfected with miR-1254-mimics and miR-1254-inhibitor lentivirus, respectively. *p < 0.05; **p < 0.01; ***p < 0.001. The data expressed as the mean ± SD

To confirm whether miR-1254 was abnormally regulated in GC tissues, 90 pairs of GC tissues and adjacent normal tissues were collected to examine the relative expression of miR-1254 by miRNA RT-PCR. As shown in Fig. 1a, compared with the paired adjacent tissues, the expression of miR-1254 was lower in human GC tissues. The expression of miR-1254 was further examined in normal gastric mucosa epithelial cells (GES-1) and GC cells lines (SGC7901, BGC823, MKN45, HGC27, MGC803) by miRNA RT-PCR. As shown in Fig. 1b, the expression of miR-1254 was lower in GC cell lines than that in GES-1 cells. Furthermore, we investigated the correlation between the miR-1254 expression and clinicopathologic features of GC. Ninety GC patients were divided into a high miR-1254 expression group and a low miR-1254 expression group according to miR-1254 expression levels whether higher than the mean expression or not. As shown in Table 1, 44 cases were in the high miR-1254 group, while 46 cases were in the low miR-1254 group. Decreased miR-1254 expression was associated with larger tumor size, poorer histological type, and lymph node metastasis. These data indicated that miR-1254 was downregulated in GC tissues and cell lines.

**Table 1:** Expression of miR-1254 and Smurf1 in human gastric cancer according to patients’ clinicopathological characteristics

Characteristics	Number	miR-1254 expression		*p*-value	Smurf1 expression		*p*-value
		High group	Low group		High group	Low group	
*Age (years)*							
<60	29	13	16	0.595	14	15	0.507
≥60	61	31	30		34	27	
*Gender*							
Male	42	23	19	0.297	23	19	0.799
Female	48	21	27		25	23	
*Size (cm)*							
<3	40	26	14	0.006*	15	25	0.007*
≥3	50	18	32		33	17	
*Histological type*							
Well-moderately	35	23	12	0.011*	13	22	0.014*
Poorly signet	55	21	34		35	20	
*Stage*							
I/II	41	23	18	0.211	18	23	0.101
III/IV	49	21	28		30	19	
*T grade*							
T1+T2	34	20	14	0.142	16	18	0.353
T3+T4	56	24	32		32	24	
*Lymph node metastasis*							
Absent (N0)	37	25	12	0.003*	14	23	0.014*
Present (N1–N3)	53	19	34		34	19	

Clinicopathological results were compared using Pearson *χ*^2^ tests

*p < 0.05 statistically significant difference

### MiR-1254 inhibits the proliferation of GC cell lines

**Fig. 2:**
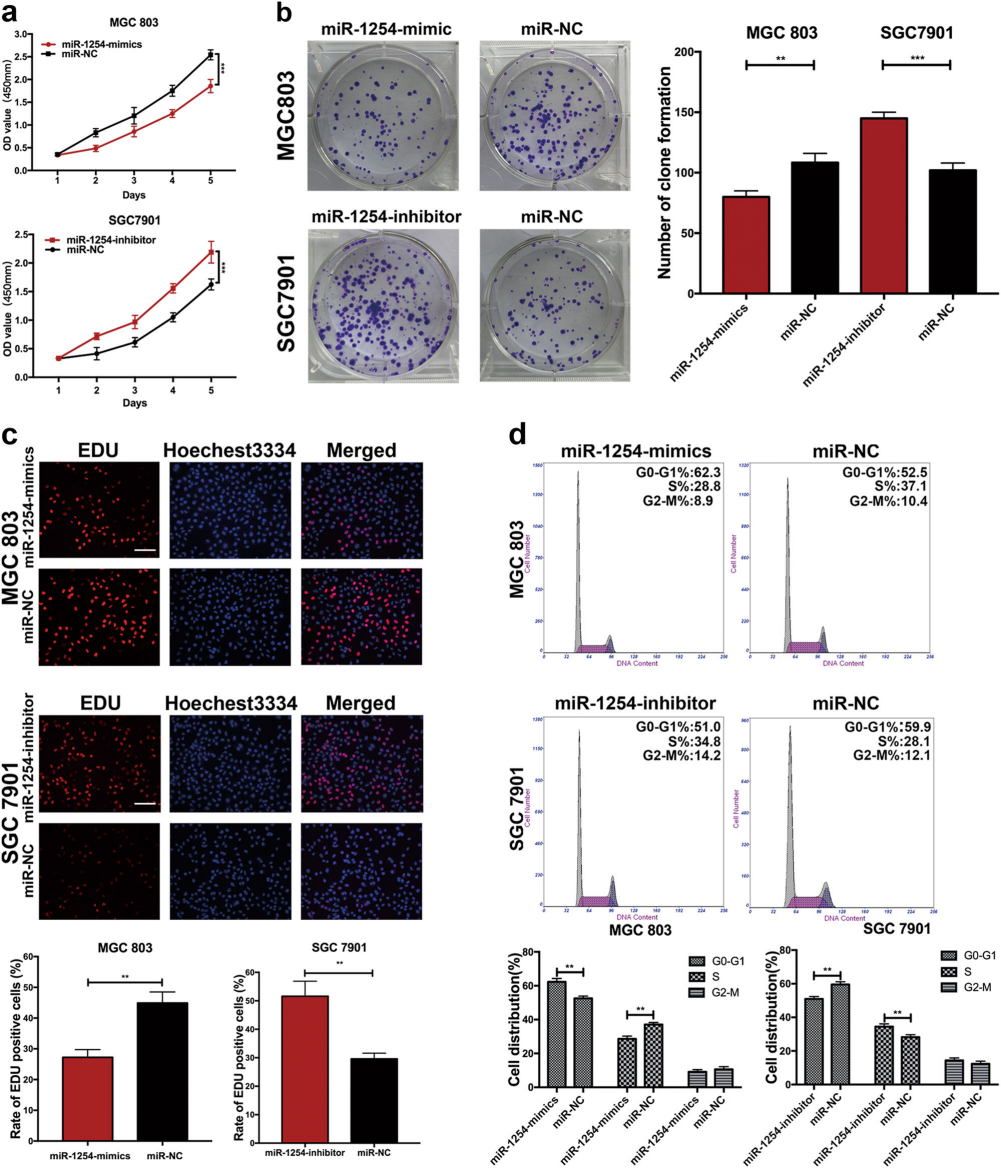
miR-1254 inhibited the proliferation of GC cells. **a** CCK-8 assay was used to explore the proliferation of GC cells transfected with miR-1254-mimics and miR-1254-inhibitor lentivirus. **b** Effects of miR-1254 difference on the colony formation of GC cells. **c** EDU incorporation assay was used to examine the effect of miR-1254 on proliferation in MGC803 cells transfected with the miR-1254 mimics and SGC7901 transfected with the miR-1254 inhibitor (scale bar: 200 μm). **d** Effects of miR-1254 difference on cell cycle distribution of GC cells. *p < 0.05; **p < 0.01; ***p < 0.001. The data expressed as the mean ± SD

To investigate the role of miR-1254 in GC. We selected MGC803 and SGC7901 cells to verify the biological function of miR-1254 according to the results of expression levels of miR-1254 in GC cells by miRNA RT-PCR. Then miR-1254 mimics and inhibitor lentivirus were constructed to infect MGC803 and SGC7901 cells, respectively. MiRNA RT-PCR was used to verify the efficiency of transfection of cell lines. As shown in Fig. 1c, miR-1254 was remarkably upregulated in MGC803-mimics and downregulated in SGC7901-inhibitor compared with the levels detected in the control groups. CCK-8 assay was used to confirm the effect of miR-1254 on the proliferative ability of GC cells. The results revealed that growth rate of MGC803 cells transfected with miR-1254 mimics was significantly decreased compared with negative control (miR-NC), while the SGC7901 cells transfected with miR-1254 inhibitor showed the opposite effect (Fig. 2a). Consistent with the results of CCK-8 assay, colony formation assay showed that over-expression of miR-1254 could suppress GC cell proliferation, whereas inhibition of miR-1254 promoted the effects (Fig. 2b). We also used the EDU incorporation assay to examine the effect of miR-1254 on proliferation. As shown in Fig. 2c, MGC803 cells transfected with the miR-1254 mimics revealed a remarkably decreased EdU-positive cells compared with control group, while SGC7901 cells with the miR-1254 inhibitor increased the positive rate. Flow cytometry was carried out to reflect the cell cycle distribution of GC cells. The results showed that MGC803 cells transfected with miR-1254 mimics extended cells cycle in the G0/G1 phase, while the opposite trend was exhibited in SGC7901 cells transfected with the miR-1254-inhibitor (Fig. 2d). In summary, the results mentioned above revealed that overexpression of miR-1254 could suppress the proliferation of GC cells in vitro.

### MiR-1254 negatively regulates the migration and invasion of GC lines

**Fig. 3:**
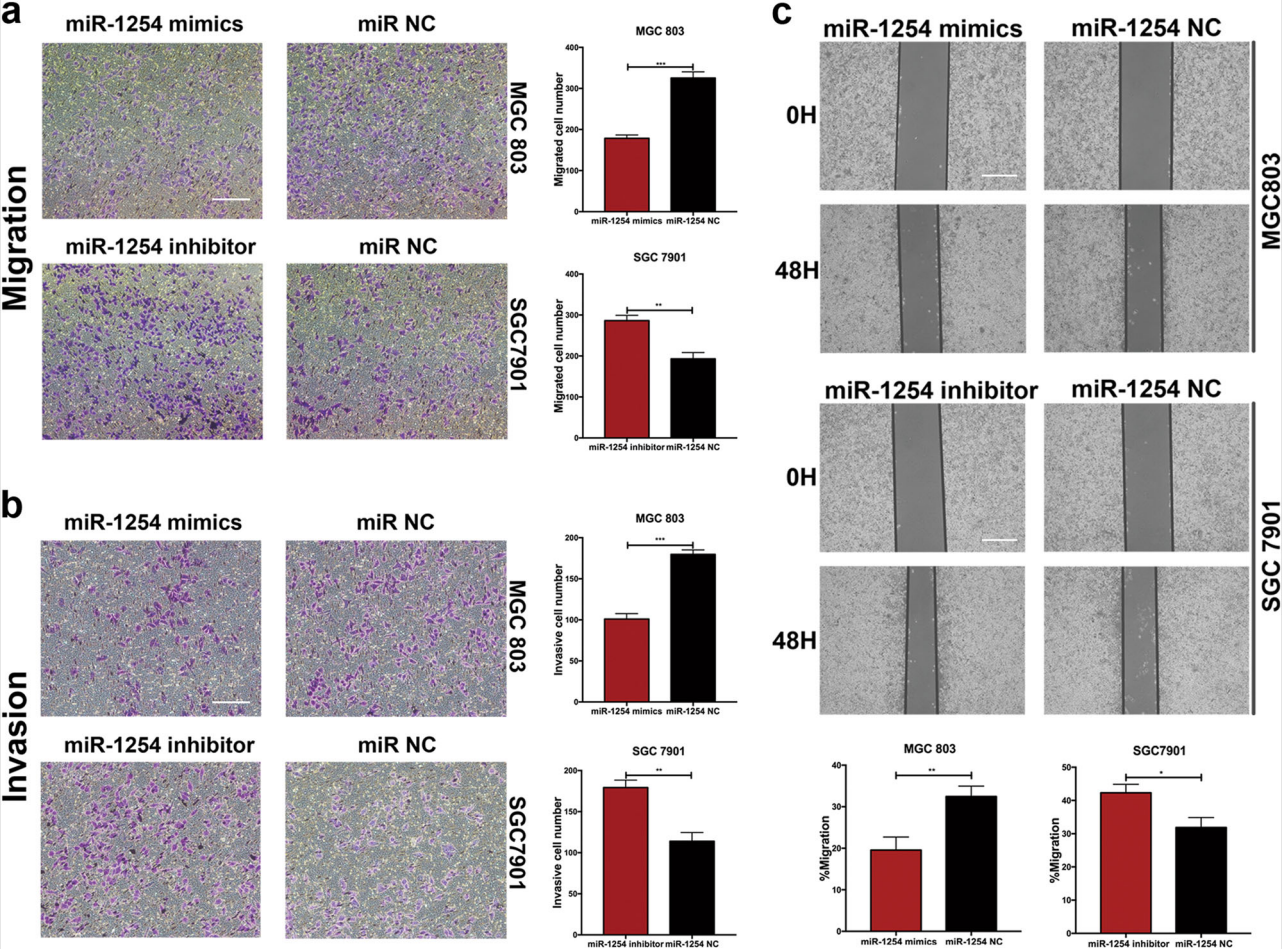
miR-1254 negatively regulated the migration and invasion of GC cells. **a** and **b** Cell migration and Matrigel invasion assays was used to examine the effects of miR-1254 on GC cell migration and invasion, the migration and invasion capability were quantified as cell numbers (scale bar: 200 μm). **c** Wound healing was performed to determine the ability of cell migration after transfected with miR-1254-mimics and miR-1254-inhibitor, the migratory rate was calculated by migration distance/original width (scale bar: 100 μm). *p < 0.05; **p < 0.01; ***p < 0.001. The data expressed as the mean ± SD

To further investigate the inhibitory role of miR-1254 in GC cells. Cell migration and Matrigel invasion assays were used to examine the effects of miR-1254 on GC cell migration and invasion. As shown in Fig. 3a, the migration of MGC803 cells transfected with miR-1254 mimics was reduced, whereas migration was enhanced by miR-1254 knockdown in SGC7901 cells. Moreover, compared with the control cells, the number of invasion cells was also decreased by miR-1254 overexpression in MGC803 cells, while knockdown of miR-1254 showed the opposite effect (Fig. 3b). In the wound healing assay, overexpression of miR-1254 reduced the migration rate of GC cells. On the contrary, knockdown of miR-1254 significantly accelerated the migration of GC cells (Fig. 3c). These results suggested miR-1254 suppressed cell migration and invasion in GC cells.

### Smurf1 is a predicted target of miR-1254

To predict the targets of miR-1254, Targetscan (http://www.targetscan.org/vert_71/), miRDB (http://www.mirdb.org), and RNA22-HAS (https://cm.jefferson.edu/rna22/Precomputed/) were employed. According to the prediction results from three miRNA bioinformatics websites, we found that Smurf1, an oncogene in many malignancies, might be one of the target genes of miR-1254.

### Smurf1 is upregulated in human GC tissues and cell lines

**Fig. 4:**
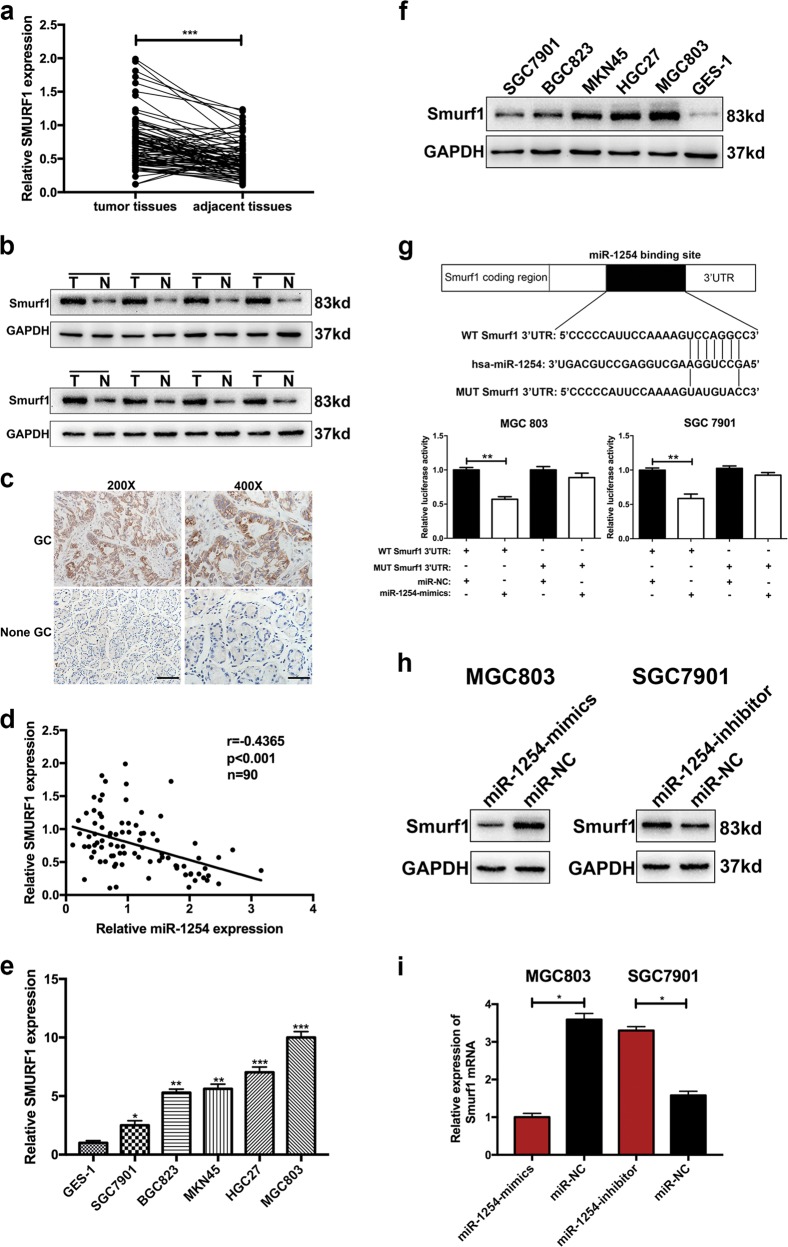
Smurf1 was up-regulated in GC tissues and cells and was confirmed to be a direct target gene of miR-1254. **a** Ninety pairs of GC tissues and adjacent normal tissues were collected to examine the relative expression of Smurf1 by qRT-PCR. **b** Western blot was used to determine Smurf1 protein expression in eight pairs GC specimens and adjacent normal tissues. **c** Immunohistochemistry staining was used to determine the protein level of Smurf1 in GC tissues and adjacent tissues (scale bar: 50 μm). **d** The correlation between the expression of miR-1254 and Smurf1. **e** and **f** The expression of Smurf1 in GC cells and GES-1 were detected by qRT-PCR and western blot, respectively. **g** Luciferase reporter assay was conducted to confirm that miR-1254 is directly bound to the 3′-UTR region of Smurf1. The result of Luciferase activity was analyzed in cells co-transfected with miR-1254-mimics or negative control with pGL3-Smurf1-WT or pGL3-Smurf1-MUT. **h** The expression level of Smurf1 protein in GC cells was verified by western blot. **i** The expression level of Smurf1 mRNA in GC cells was detected by qRT-PCR. *p < 0.05; **p < 0.01; ***p < 0.001. The data expressed as the mean ± SD

To evaluate the relation between miR-1254 and Smurf1 in GC. Ninety pairs of GC tissues and adjacent normal tissues were collected to examine the relative expression of Smurf1 by qRT-PCR. As shown in Fig. 4a, the expression of Smurf1 was upregulated in GC tissues compared to normal tissues. Then western blot was used to determine Smurf1 protein expression in randomized eight pairs GC specimens and adjacent normal tissues from 90 pairs specimens. As shown in Fig. 4b, Smurf1 expression was higher in GC tissues than in adjacent normal tissues. Consistently, immunohistochemistry also showed the same result as the western blot (Fig. 4c). Notably, miR-1254 expression levels were negatively associated with Smurf1 expression (Fig. 4d). Next, Smurf1 expression was detected in GC cells and GES-1 with qRT-PCR and western blot. The results showed that the relative expression of Smurf1 both on mRNA and protein level in GC cells was higher than that in GES-1 (Fig. 4e and f). Moreover, we analyzed the correlation between Smurf1 expression levels and clinicopathological feature. As shown in Table 1, high Smurf1 expression was associated with larger tumor size, poorer histological type and lymph node metastasis. These results revealed that Smurf1 might be a direct targets of miR-1254.

### The Sumrf1 3′-UTR is a target of miR-1254

Dual luciferase reporter assays were used to further confirm that Smurf1 was a direct target of miR-1254. Wild-type (WT) and mutant-type (MUT) Smurf1 3′-UTR sequences (the later containing site-directed mutations) were cloned into pGL3 reporter plasmids. miR-1254 mimics and pGL3-WT-Smurf1 3′-UTR were co-transfected both in MGC803 and SGC7901 cells. We observed a significantly decreased luciferase activity by comparison with control group. Instead, overexpression of miR-1254 did not affect luciferase activity in the cell transfected with pGL3-MUT-Smurf1 3′-UTR (Fig. 4g). All these results suggested that the 3′-UTR of Smurf1 was targeted by miR-1254 and that the point mutations in this sequence abolished this interaction

### MiR-1254 represses Smurf1 protein expression by mRNA degradation

We confirmed that the 3′-UTR of Smurf1 was targeted by miR-1254. To verify specifically the mechanism by which miR-1254 regulated Smurf1, western blot was used to investigate the Smurf1 protein expression levels. Compared with the control group, Smurf1 protein levels were evidently reduced in MGC803 cells after transfection with miR-1254 mimics. However, Smurf1 protein levels were increased in SGC7901 cells transfected with the miR-1254 inhibitor (Fig. 4h). Then, qRT-PCR was used to observe the Smurf1 mRNA levels in MGC803 cells and SGC7901 cells. As shown in Fig. 4i, Smurf1 mRNA expression levels were lower in MGC803 after transfected with the miR-1254 than in negative control. In contrast, the relative expression of smurf1 mRNA transfected with miR-1254 inhibitor was higher than the control. The results of dual luciferase reporter assays and qRT-PCR suggested that miR-1254 repressed Smurf1 protein expression by mRNA degradation.

### MiR-1254 inhibits proliferation, migration, and invasion in GC cells by targeting Smurf1

**Fig. 5:**
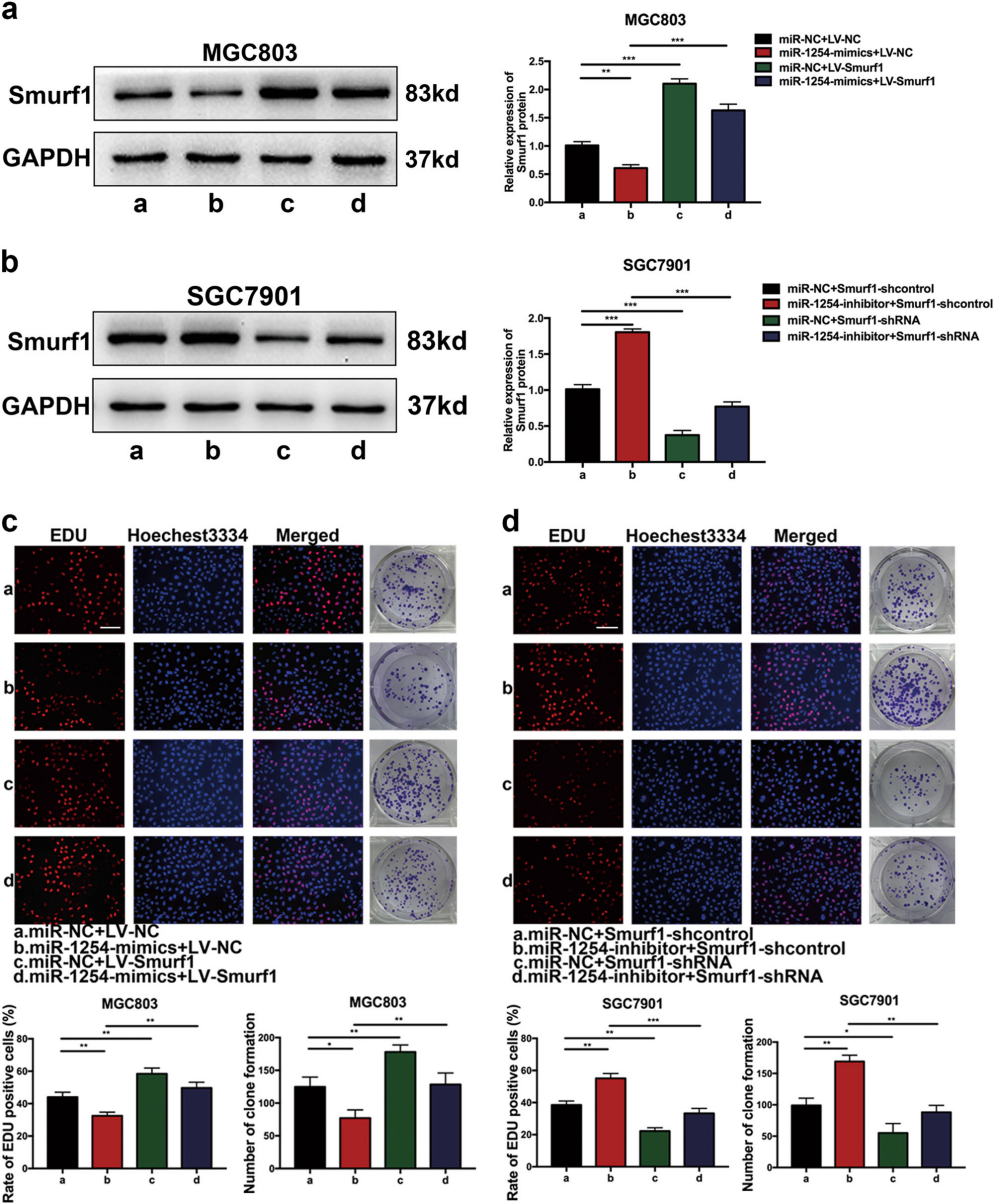
Overexpressed Smurf1 could partially reverse the effects of miR-1254 on GC cells. **a** and **b** Western blot was used to verify the expression of Smurf1 in each group. **c** EDU incorporation assay and colony formation assay were conducted to verify that ectopic Smurf1 expression could reverse proliferation induced by miR-1254 overexpression in MGC803 cells (scale bar: 200 μm). **d** Similar rescue experiments for miR-1254 silencing was performed by downregulation of Smurf1 in SGC7901 cells. *p < 0.05; **p < 0.01; ***p < 0.001. The data expressed as the mean ± SD

**Fig. 6:**
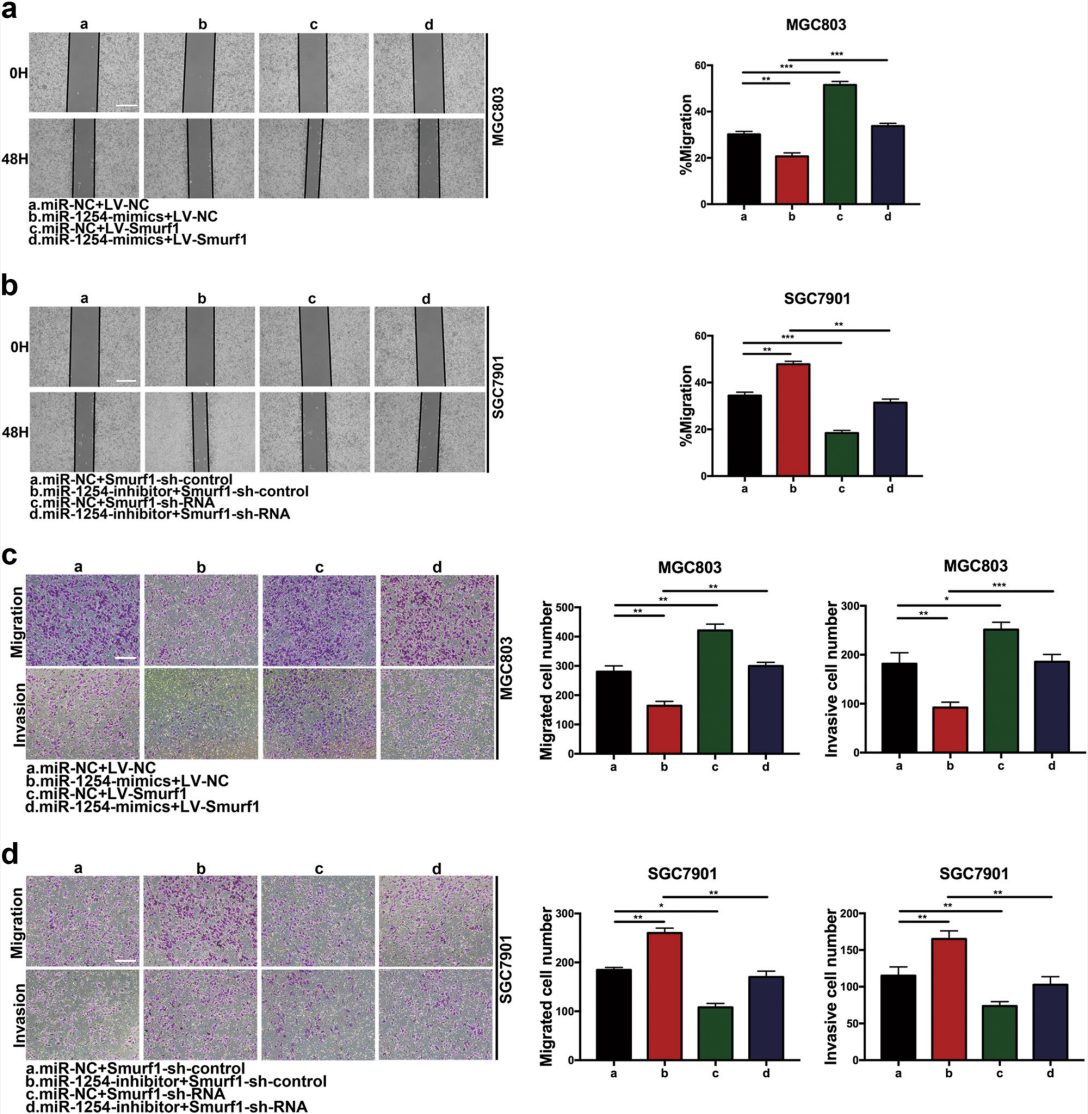
Overexpressed Smurf1 could partially reverse the effects of miR-1254 on GC cells. **a** and **b** The change of cell migration was examined by wound healing assay in MGC803 cells and SGC7901 cells (scale bar: 100 μm). **c** and **d** Transwell assay was carried out to confirm the effects of Smurf1 alteration in migration and invasion of GC cells (scale bar: 200 μm). *p < 0.05; **p < 0.01; ***p < 0.001. The data expressed as the mean ± SD

We demonstrated that overexpression of miR-1254 inhibited proliferation, migration, and invasion in GC cells and inhibited Smurf1 protein expression by mRNA degradation. In contrast, the opposite result was carried out by miR-1254 knockdown. To further confirm that whether miR-1254 inhibited proliferation, migration, and invasion in GC by modulating Smurf1, the vector LV-Smurf1 (only contains the Smurf1 coding sequence and does not contain the miR-1254 target sequence) was first constructed to allow Smurf1 expression. Then, MGC803 cells were co-transfected with miR-NC+LV-NC, miR-1254-mimics+LV-NC, miR-NC+LV-Smurf1, and miR-1254-mimics+LV-Smurf1. Western blot was used to examine the Smurf1 protein expression in MGC803 cells (Fig. 5a). Through EDU incorporation assay, colony formation assay, wound healing assay, and transwell assay, we have found that ectopic Smurf1 expression could reverse the suppression of proliferation, migration, and invasion induced by miR-1254 overexpression in MGC803 cells (Figs. 5c, 6a and c). Similarly, smurf1 protein expression levels in SGC7901 cells were confirmed by western blot analysis (Fig. 5b). The effects of miR-1254 knockdown were counteracted by Smurf1 downregulation in SGC7901 cells (Figs. 5d, 6b and d). These findings suggested that miR-1254 inhibited GC cells proliferation, migration, and invasion by targeting Smurf1 directly.

### MiR-1254 suppresses the growth of GC in vivo

**Fig. 7:**
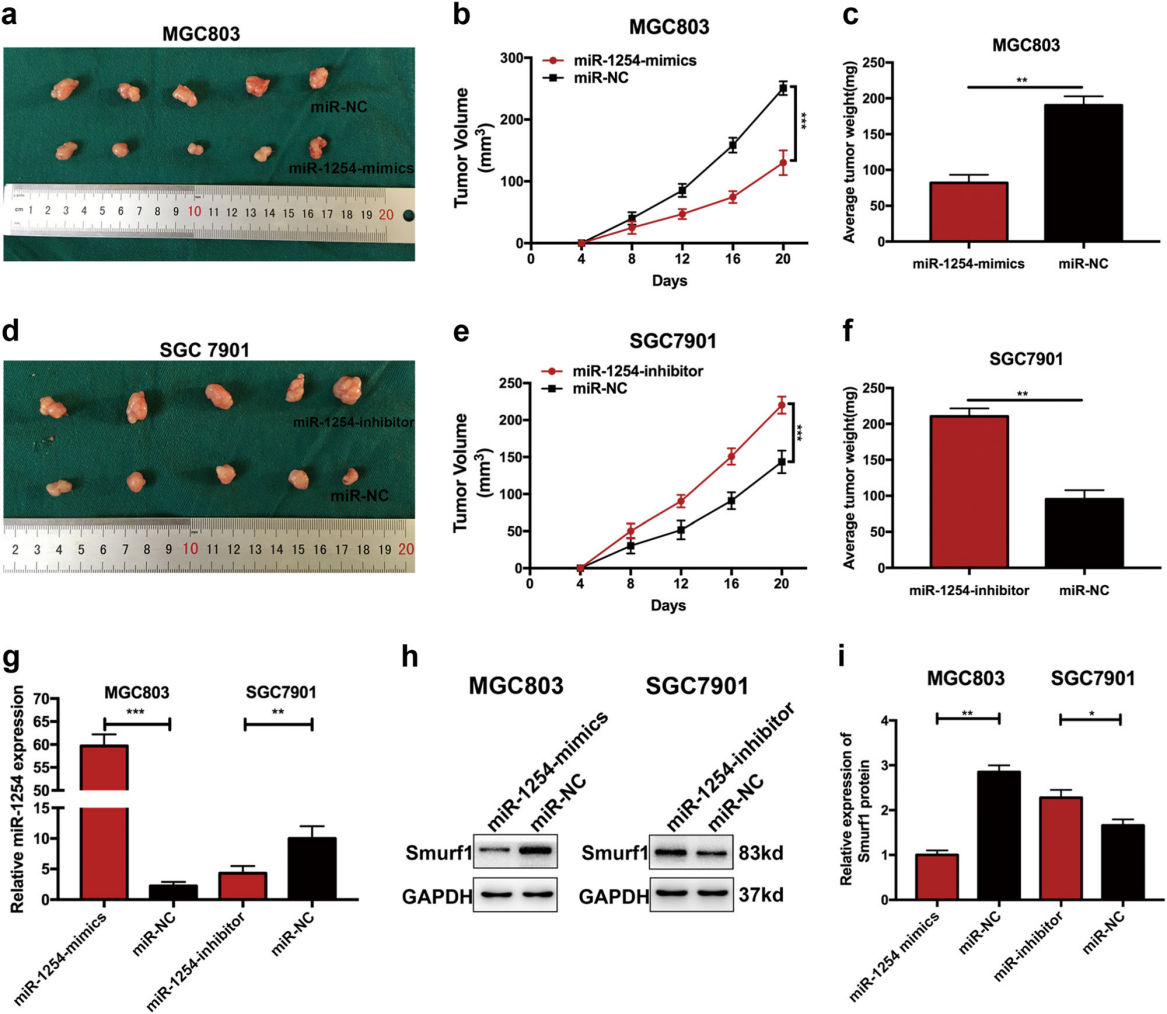
miR-1254 inhibited tumorigenicity in vivo. **a**, **d** Xenograft tumors were obtained from different groups of nude mice transfected with miR-NC, miR-1254-mimics, and miR-1254-inhibitor, respectively. **b**, **c**, **e**, **f** Tumor growth curve and tumor weight were significantly different between miR-1254-mimics or miR-1254-inhibitor and corresponding control group. **g** The expression levels of miR-1254 in the implanted tumors that were transfected with miR-1254-mimics and miR-1254-inhibitor were explored by miRNA RT-PCR. **h** and **i** The expression of Smurf1 protein level were determined by western blot. *p < 0.05; **p < 0.01; ***p < 0.001. The data expressed as the mean ± SD

To demonstrate the effects of miR-1254 on tumor growth in vivo, cells transfected with miR-1254-mimics or miR-1254-inhibitor were injected into flanks of nude mice, and cells transfected with miR-NC were as negative control. As shown in Fig. 7a–c, miR-1254-mimics group exhibited a significant decrease in tumor volume and weight compared with control group. Conversely, compared with control group, the tumor volume and weight were increased in miR-1254-inhibitor group (Fig. 7d–f). Furthermore, the miRNA RT-PCR analysis revealed high miR-1254 expression in the miR-1254-mimics group, while reduced expression was demonstrated in miR-1254-inhibitor group (Fig. 7g). Western blot was used to investigate the Smurf1 protein expression levels. Compared with the control group, Smurf1 protein levels were obviously reduced in miR-1254-mimics group. However, Smurf1 protein levels were promoted in miR-1254-inhibitor group (Fig. 7h and i). In summary, miR-1254 suppressed the growth of GC in vivo.

### Overexpression of miR-1254 inhibits epithelial–mesenchymal transition (EMT) by Smurf1

**Fig. 8:**
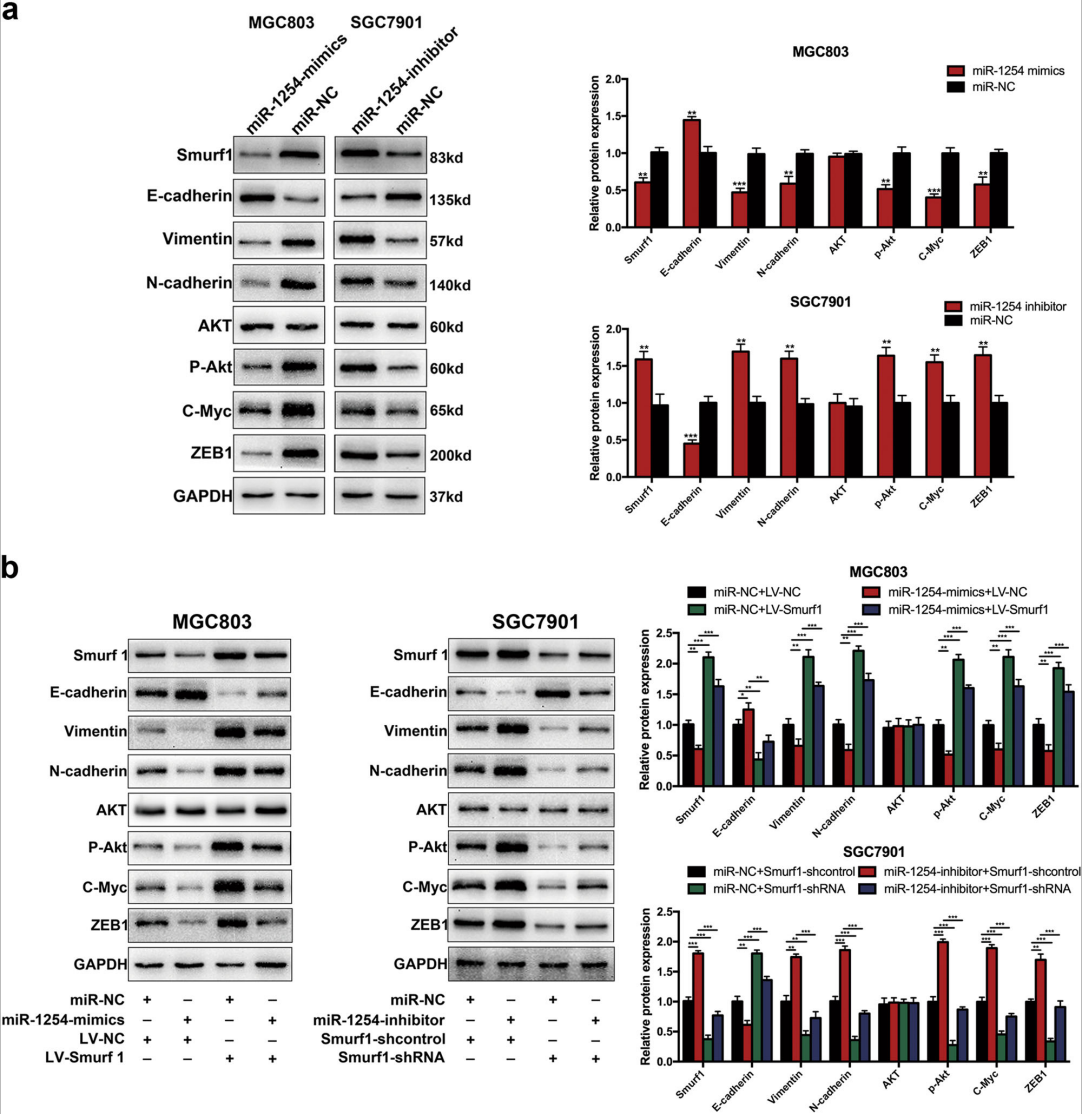
miR-1254 inhibited EMT and negatively regulated PI3K/AKT signaling pathway in GC cells by downregulation of Smurf1. **a** Western blot showed the change of biomarkers, such as E-cadherin, Vimentin, N-cadherin in EMT and AKT, p-Akt c-Myc, ZEB1 in cells transfected with miR-NC, miR-1254-mimics, and miR-1254-inhibitor. **b** The expression of these proteins were examined by western blot after the restoration of Smurf1. *p < 0.05; **p < 0.01; ***p < 0.001. The data expressed as the mean ± SD

EMT is a vital biological process that enhance tumor cells invasion and metastasis. We also have demonstrated that miR-1254 could suppress migration and invasion in GC cells by targeting Smurf1. Therefore, we evaluated the protein levels of EMT-related factors to analyze the relationship between miR-1254 and EMT. Western blot data showed that E-cadherin levels of MGC803 cells transfected with miR-1254 mimics were increased, while Vimentin and N-cadherin levels were reduced (Fig. 8a). Moreover, the MGC803 cells, co-transfected with LV-Smurf1 and miR-1254-mimics, could mitigate this change (Fig. 8b). Silencing miR-1254 expression indicated the decrease of E-cadherin and the increase of Vimentin and N-cadherin (Fig. 8a). Also, these effects could partially be recovered by co-transfecting with Smurf1-shRNA and miR-1254 inhibitor in SGC7901 cells (Fig. 8b). These data suggested overexpression of miR-1254 could inhibit EMT by Smurf1.

### MiR-1254 decreases the PI3K/AKT signaling pathway by downregulation of Smurf1

In order to detect the mechanisms how miR-1254 and Smurf1 inhibited the proliferation, migration, and invasion in GC, we investigated whether these effects were mediated by activating the PI3K/AKT signaling pathway. Western blot was used to examine Smurf1 expression levels and downstream protein AKT, P-Akt, c-Myc, and ZEB1. As shown in Fig. 8a, overexpressed miR-1254 caused a significant decrease of P-Akt, c-Myc, and ZEB1 in GC cells by comparison to control group and knockdown of miR-1254 showed the opposite effects. However, there was no significant difference in AKT expression. These effects could partially be recovered by upregulating the Smurf1 expression in miR-1254-mimics cells (Fig. 8b). Similarly, the same results can be obtained by downregulating the Smurf1 expression in miR-1254-inhibitor cells (Fig. 8b). These findings indicated that Smurf1 functioned as a facilitator in PI3K/AKT-signaling pathway. All these results suggested that Smurf1 was a downstream functional regulator of miR-1254 through PI3K/AKT-signaling pathway.

## Discussion

The aberrant expression of miRNAs is related to the development and progression of cancer^20,21^. It has been shown that miRNAs act as carcinogenic factors or tumor suppressor factors according to the specific function of targeting mRNA^22,23^. Only a few studies have reported miR-1254 is aberrantly expressed and plays special roles in different malignancies like lung cancer, thyroid cancer, and colorectal cancer^11–13^. However, the function of miR-1254 in GC and the potential molecular mechanism remain to be elucidated. In this study, we found that miR-1254 expression was obviously lower in GC tissues and cell lines than normal tissues and GES-1 cells. On the basis of a series of experiments, overexpression of miR-1254 suppressed proliferation, migration, invasion, EMT in vitro, and tumorigenicity in vivo, showing that miR-1254 may be a potential therapeutic target for GC patients. Furthermore, our research also indicated that miR-1254 decreased the PI3K/AKT signaling pathway by downregulation of Smurf1.

To clarify the mechanisms underlying the effects of miR-1254 on proliferation, migration, and invasion. Bioinformatics analysis was used to predict putative targets of miR-1254 in GC cells. Among the candidate target genes, we concentrated on Smurf1. Smurf1, a well-known E3 ubiquitin ligase, contains a phospholipid-binding C2 domain, a catalytic HECT domain, and two tandem WW domains and each domain has its own unique function^24,25^. Smurf1 has been reported to involve in the development and progression of numerous malignancies^26,27^. Previous studies have revealed that dysregulated Smurf1 was mainly a facilitator in various cancers^17,18^. However, some other studies showed that Smurf1 existed as the role of a suppressor in malignancies^28,29^. In our study, we examined the expression of Smurf1 between GC tissues and paired adjacent normal tissues, as well as in GC cell lines and GES-1 cells. The results revealed that Smurf1 expression in GC tissues was much higher than that in paired normal tissues and GES-1 cells. The higher expression of Smurf1 was also found to be associated with larger tumor size, poorer histological type, and lymph node metastasis. Furthermore, the dual-luciferase reporter assays confirmed that miR-1254 directly bound to its 3′UTR and overexpression of miR-1254 could suppress Smurf1 expression by degrading Smurf1 mRNA. Moreover, ectopic expression of Smurf1 partially counteract the effects of the suppression of proliferation, migration, and invasion caused by miR-1254 overexpression. In summary, the results of our study demonstrated that the inhibitory effects of miR-1254 in GC was mediated by downregulation of Smurf1.

EMT refers to the biological process by which epithelial cells are transformed into specific stromal phenotype cells through specific procedures^30,31^. In cancer, the EMT is associated with tumor stemness, metastasis, and resistance to therapy^32^. With the development of cancer, the epithelial cells gradually lose their connection with the basement membrane^33^. EMT is a complex cellular process that regulates transformations in cell morphology and function during the development and progression of cancer. According to the previous research, EMT occurs through distinct intermediate states, moreover, spontaneous EMT in primary TCs in vivo proceeds through distinct intermediate states with different invasive, metastatic, and differentiation characteristics^34^. Furthermore, EMT is typically characterized by decreased expression of cell adhesion proteins, such as E-cadherin and cytokeratins, while it is increased in mesenchymal-associated molecules, such as N-cadherin and vimentin^35^. Thus, EMT plays an important role in cancer migration and invasion. Lately, increasing studies have confirmed miRNAs were related to EMT in malignancies, Yu et al. reported that miR-190 suppressed breast cancer metastasis by regulating TGF-β-induced EMT^36^. In hepatocellular carcinoma, Guo et al. found miR-429 suppressed tumor migration and invasion by targeting CRKL via inhibiting Raf/MEK/ERK pathway and EMT^37^. In this study, we verified that overexpressed miR-1254 could suppress EMT by dramatically increasing levels of E-cadherin, while upregulating the expression of N-cadherin and Vimentin. These findings were consistent with our migration and invasion results.

Previous studies have shown that the phosphatidylinositol-3-kinase (PI3K)/AKT-signaling pathway was associated with tumor proliferation and metastasis^38^. It has been reported that Smurf1 is a regulator of PI3K/AKT-signaling pathway^19,39^. Furthermore, the downstream protein of PI3K/AKT-signaling pathway, such as c-Myc and ZEB1 could regulate proliferation, migration, and invasion in cancer cells^40,41^. Therefore, we investigated the involvement of the PI3K/AKT-signaling pathway in the effects of alterations in miR-1254 expression in GC. The results showed that miR-1254 decreased the PI3K/AKT-signaling pathway by downregulation of Smurf1. Moreover, miR-1254 acted as a negative regulator of PI3K/AKT-signaling pathway to inhibit proliferation, migration, and invasion of GC. However, the results of this study did not rule out the possibility that other signaling pathways may also be affected by miR-1254.

With the development of miRNAs, they could provide a valid option for the clinical treatment of specific malignancies in the future^42,43^. Both tumor suppressive or oncogenic miRNAs are suited for therapeutic targets, and they are negatively correlated expression with their target gene^44^. According to related reports, miRNA-targeted therapeutics have reached clinical development, including a mimic of the tumor suppressor miRNA miR-34, which reached phase I clinical trials for treating cancer, and antimiRs targeted at miR-122, which reached phase II trials for treating hepatitis^45^. In the present study, we revealed that miR-1254 inhibits the proliferation, migration, and invasion in GC. It may become a new potential target for the treatment of GC in the future.

In summary, our data demonstrated that miR-1254 was down-regulated in GC. MiR-1254 was correlated with tumor size, histological type, and lymph node metastasis and that overexpression of miR-1254 inhibited GC proliferation, migration, invasion, and EMT. Moreover, miR-1254 downregulated Smurf1 expression through directly binding to the Smurf1 3′-UTR. We also found that PI3K/AKT-signaling pathways played an important role in this process. In general, our findings indicated the miR-1254 could be a potential therapeutic target for GC.

## Materials and methods

### Tissue samples

Human GC tissues and paired adjacent normal tissues were collected from 90 patients with GC who underwent radical gastrectomy at the Department of General Surgery, The First Affiliated Hospital of Nanjing Medical University (NMU), China. After surgical resection, specimens were rapidly frozen in liquid nitrogen for experiments need. Histopathologic diagnoses were carried out by experienced pathologists. This study was approved by the Ethics Committee of the First Affiliated Hospital of NMU. Written informed consent was signed before specimen collection.

### GC cell lines

The Human GC cell lines, SGC7901, BGC823, MKN45, HGC27, MGC803, and the normal human gastric mucous epithelium cell line GES-1 were purchased from the American Type Culture Collection (Manassas, VA, USA). Cells were cultured in RPMI-1640 medium supplemented with 10% fetal bovine serum (WISENT, Canada) and 1% antibiotics (100 U/ml penicillin G and 100 mg/ml streptomycin). They were incubated in a humidified atmosphere at 37 °C containing 5% CO_2._

### Quantitative real-time polymerase chain reaction (qRT-PCR)

Total RNA was extracted from GC tissues and cells using TRIzol reagent (Invitrogen, Carlsbad, CA, USA) according to the manufacturer’s recommendations. Then we used PrimeScript RT Reagent (TaKaRa, Japan) to synthesize cDNA. qRT-PCR was performed on a 7500 Real-time PCR System (Applied Biosystems, Carlsbad, CA, USA) with AceQ qPCR SYBR® Green Master Mix (Vazyme, Biotech, Co.,Ltd). The level of β-actin expression was used as the internal control for Smurf1. The primers employed in this study were as follows: Smurf1 forward, 5′-CTGGATGCTTTTGGTCTGGT-3′ and Smurf1 reverse, 5′-CCTGATAGACGCGAACACAG-3′; β-actin forward, 5′-GCATCGTCACCAACTGGGAC-3′ and β-actin reverse, 5′-ACCTGG CCGTCAGGCAGCTC-3′. All procedures were carried out in triplicate and relative expression was calculated by the 2^−ΔΔCT^ method.

### miRNA RT-PCR

Total RNA was extracted as mentioned above. We used the Hairpin-it^TM^ miRNA qPCR Quantitation Kit (GenePharma, China) to perform target-specific reverse transcription and the TaqMan miRNA assay. The particular primers are as follows: hsa-miR-1254 forward, 5′-AGCCTGGAAGCTGGAGCCTGCAGT-3′; Universal, 5′-GCGAGCACAGAATTAATACGAC-3′; U6 forward, 5′-CTCGCTTCGGCAGCACA-3′; U6, reverse: 5′-AACGCTTCACGAATTTGCGT-3′. We designed the snRNA U6 as the control for miR-1254 to calculate the relative expression levels of miR-1254 in respective sample using the 2^−ΔΔCT^ method. All procedures were also performed in triplicate.

### Vector and lentivirus production and cell transfection

Following the experimental design, commercially available lentiviral vectors were used to construct the LV2-hsa-miR-1254-mimic vector (miR-1254-mimic) and the LV2-hsa-miR-1254-inhibitor vector (miR-1254-inhibitor) (GenePharma, Shanghai, China). These structures were used to overexpress or knockdown miR-1254 in GC cells after being verified by DNA sequencing. The LV2 empty lentiviral construct (miR-NC) acted as a negative control. When MGC803 and SGC7901 grew to 40–50% confluence, they were infected by miR-1254-mimic, miR-1254-inhibitor, and miR-1254-NC at a suitable multiplicity of infection (MOI). Stable cell lines were obtained by using 5 μg/ml puromycin (Sigma, Aldrich) for about a week. Then we used Hairpin-itTM miRNA qPCR Quantitation Kit (GenePharma, China) to analyze the miR-1254 expression of cells. Lentiviral vector containing Smurf1 and shRNA coding sequence (LV-Smurf1, Smurf1-shRNA) were also constructed by Genepharma Biotech (Shanghai, China). The scrambled lentiviral construct was performed as a negative control. Stable cells were generated by means of the above procedure. Then we used qRT-PCR and western blot to analyze the expression of Smurf1.

### Dual luciferase reporter assay

Sequences containing WT or mutant miR-1254-binding site in the 3′-UTR of Smurf1 were synthesized by GeneScript (Nanjing, China) and cloned into the FseI and XbaI restriction sites of the pGL3 luciferase control reporter vector (Promega, USA) to acquire the Smurf1 3′-UTR reporter constructs (pGL3-WT-Smurf1 and pGL3-MUT-Smurf1). Cells in 24-well plates were co-transfected with miR-1254 mimic or control, pGL3-WT-Smurf1, and pGL3-MUT-Smurf1 using Lipofectamine 2000 (Invitrogen). Renilla luciferase expression plasmids were also transfected into the cells to act as a reference control. After transfection for 48 h, dual-luciferase reporter assay system (Promega, USA) was used to detect the Firefly and Renilla luciferase activities according to the manufacturer’s instructions.

### Western blot assay

The proteins were extracted from GC cells and tissues using RIPA lysis buffer (Beyotime, Shanghai, China), separated by sodium dodecyl sulfate polyacrylamide gel electrophoresis (SDS–PAGE) and then transferred to a polyvinylidene fluoride (PVDF) membrane. Membranes were blocked with 5% no-fat milk in TBST at room temperature for 2 h, and incubated with the specific primary antibodies overnight for 4 °C. The membranes were incubated in secondary antibodies at room temperature for 2 h. TBST was used to wash the membranes before and after incubating membranes in secondary antibodies. Proteins were detected using an enhanced chemiluminescence (ECL) detection system. GAPDH was acted as an internal control.

### Cell proliferation assay

Cell Counting Kit-8 (CCK-8) (Beyotime, Shanghai, China) was used to measure cell proliferation according to the manufacturer’s recommendations. Cells in 96-well plates (1 × 10^3^ cells/well) were cultured in RPMI 1640 containing 10% FBS for 5 days. At a fixed time of day, CCK8 reagent was added to corresponding plate and cells were incubated for 2 h at 37 °C. The absorbance was detected at 450 nm and was used to assess the capability of cell proliferation.

### 5-Ethynyl-2′-deoxyuridine (EdU) assay

The EdU assay kit (RiboBio, China) was used to measure cell proliferation. The cells were cultured in 96-well plates (5 × 10^3^ cells/well) with RPMI 1640 (10% FBS) for 24 h. On the second day, cells were incubated with 50 μM EdU for 2 h at 37 °C and fixed in 4% formaldehyde for 30 min. Glycine is used to neutralize formaldehyde to ensure the dyeing reaction system. After permeabilizing with 0.5% TritonX-100 for 10 min at room temperature, 1 × Apollo^®^ reaction cocktail (100 μl) was used to react with the EdU for 30 min. Finally, 1 × Hoechst (100 μl) was used to stain the nuclei. Cells were visualized under fluorescence microscope (Nikon, Japan).

### Colony formation assay

Stable transfected GC cells were plated in six-well plate (500 cells/well) and cultured with RMPI-1640 medium for about 2 weeks. Proliferating colonies were stained with 1% crystal violet (Beyotime, Shanghai, China). The colonies were counted and photographed after containing 50 cells or more. All procedures were performed in triplicate.

### Flow cytometric (FCM) analysis of cell-cycle

FCM was used to measure cell-cycle. The transfected cells were fixed in 75% ethanol before storing at −20 °C overnight. After that, the cells were incubated with RNAse, and stained with PI-staining solution (500 μl, MultiSciences, China) for 15 min at room temperature for cell-cycle analysis.

### MGC803 and SGC7901 migration and invasion assays

The migratory and invasive ability of cells were assayed by using a 6.5 mm chamber with 8 μm pores (Corning Costar Corp., USA). To investigate the migratory ability of cells, 2 × 10^4^ stable transfected GC cells were suspended in 200 μl serum-free RMPI-1640 medium and plated in the top chamber. Next, 500 μl RMPI-1640 medium with 10% fetal bovine serum was added to the lower chamber of the well and the cells were incubated under chemotactic drive at 37 °C for 24 h. The cells were stained with 1% crystal violet for 30 min and cells on the upper surface of membrane were removed by cotton swabs. We counted and imaged the cells on the bottom surface of membrane by microscope (Olympus Corp. Tokyo, Japan) at proper magnification in four random fields. For invasion assays, we added 0.1 ml Matrigel (50 μg/ml, BD Biosciences, USA) onto the plate surface and incubated for 2 h, and remaining operating steps were consistent with mentioned above. The experiments were performed in triplicate.

### Wound healing assay

Wound healing assay was used to further investigate cell migration. Stable transfected GC cells (5 × 10^5^) were seeded in six-well plate. Linear scratch wounds were created by 200 μl sterile pipette tip when each well was filled with cells. Next, the plate was washed by PBS for several times to remove the suspended cell and the cells were cultured in serum-free media. After 0 and 48 h, we imaged the wounds at the same position under the microscope and the distance between the wound sides was calculated. Experiments were performed in triplicate.

### Immunohistochemistry

All specimens containing GC tissues and paired adjacent normal tissues were fixed in 4% formalin and then embedded in paraffin. After blocking endogenic peroxides and proteins, 4 μm sections were incubated overnight at 4 °C with primary antibodies specific for Smurf1 and Ki-67 (Abcam). After washing with PBS, slices were incubated in HRP-polymer-conjugated secondary antibody at 37 °C for 1 h and then stained with the 3,3-diaminobenzidine (DAB) solution for 3 min to visualize the staining. The nuclei were counterstained with hematoxylin. Tumor slices were inspected in a blinded manner. We selected three random fields to observe the percentage of positive tumors and cell-staining intensity.

### Tumor xenograft in animals

Four-week-old female BALB/c nude mice were purchased from Animal Center of NMU, and all experimental animals were in accordance with NMU Institutional Animal Care and Use Committee. A total of 20 female nude mice were randomly allocated to four groups, and MGC803-miR-1254-mimics, MGC803-miR-NC, SGC7901-miR-1254-inhibitor, and SGC7901-miR-NC stable cells (1 × 10^6^ cells/100 μl PBS) were injected into the flanks of the nude mice in the respective groups. The tumor volume was measured with Vernier calipers every 4 days and was calculated by the formula: volume = (length × width^2^)/2. Finally, the mice were euthanized after 3 weeks.

### Statistical analysis

Each experiment was repeated three times and the data of experiments were shown as mean ± standard deviation (SD). Statistical analyses were performed using SPSS v19.0. Clinicopathological results were compared using Pearson *χ*^2^ tests. Analysis of variance (ANOVA) was used to compare the treated group and control group. *P* < 0.05 was considered to be statistically significant.
